# B Cell Lymphoma 6 (BCL6): A Conserved Regulator of Immunity and Beyond

**DOI:** 10.3390/ijms252010968

**Published:** 2024-10-11

**Authors:** Clifford Liongue, Farooq L. J. Almohaisen, Alister C. Ward

**Affiliations:** 1 School of Medicine, Deakin University, Geelong, VIC 3216, Australia; c.liongue@deakin.edu.au (C.L.); farooq.almohaisen@stu.edu.iq (F.L.J.A.); 2 Institute for Mental and Physical Health and Clinical Translation, Deakin University, Geelong, VIC 3216, Australia; 3 Department of Medical Laboratory Techniques, Southern Technical University, Basra 61001, Iraq

**Keywords:** BCL6, B cell, cancer, germinal center, lymphoma, macrophage, repressor, T cell, transcription factor

## Abstract

B cell lymphoma 6 (BCL6) is a conserved multi-domain protein that functions principally as a transcriptional repressor. This protein regulates many pivotal aspects of immune cell development and function. BCL6 is critical for germinal center (GC) formation and the development of high-affinity antibodies, with key roles in the generation and function of GC B cells, follicular helper T (Tfh) cells, follicular regulatory T (Tfr) cells, and various immune memory cells. BCL6 also controls macrophage production and function as well as performing a myriad of additional roles outside of the immune system. Many of these regulatory functions are conserved throughout evolution. The *BCL6* gene is also important in human oncology, particularly in diffuse large B cell lymphoma (DLBCL) and follicular lymphoma (FL), but also extending to many in other cancers, including a unique role in resistance to a variety of therapies, which collectively make BCL6 inhibitors highly sought-after.

## 1. Introduction

The B cell lymphoma 6 (BCL6, also called BCL6A) protein was first identified as the product of a gene involved in chromosomal translocations in the context of non-Hodgkin’s lymphoma (NHL) [1]. This protein has subsequently been identified as a multi-functional regulator with a conserved structure and function. BCL6 is particularly important in immune cell development, which is largely mediated by its strong transcriptional repressor functions. This includes its pre-eminent roles in germinal center (GC) reactions [2], being required for the formation and development of GC B cells [3] and the differentiation and maintenance of follicular helper T (Tfh) cells [4,5]. Its function also extends to a range of other immune cells, including other helper T (Th) and specific regulatory T (Treg) and memory T (Tm) cell populations [6,7,8], as well as macrophages [9,10]. Diverse additional roles have also been identified outside of the immune system. A key aspect of BCL6’s function is its impact on other transcriptional regulators, with the disruption of such regulatory networks linked to various malignancies [11]. This review provides an overview of BCL6 and its role in the normal development and function of various immune cells and other populations, as well as how this is conserved across diverse species. It also examines the contribution of BCL6 to the etiology of lymphomas and other hematological malignancies and solid tumors, with a consideration of its role in therapy resistance and its application as a therapeutic target.

## 2. Gene Conservation and Evolution

BCL6 homologues are highly conserved. Human BCL6 shares around 95% identity with mouse BCL6 [12] and approximately 60% identity with Bcl6 proteins from teleost fish, such as pufferfish and zebrafish [13,14], indicative of conserved function across vertebrates. BCL6 is highly related to the BCL6B protein, which is also conserved across a wide range of vertebrate species [15]. This extends to teleost fish, which possess Bcl6b and an additional related protein termed Bcl6ab [13,14]. Invertebrates also harbor proteins related to BCL6, typified by the fruit-fly Ken and Barbie (Ken) protein, which contains a subset of functional domains [16,17]. This suggests an evolutionary model in which a precursor of both the *BCL6* and *ken* gene lineages existed in the common ancestor of invertebrates and vertebrates, which evolved into a precursor of the ken-related genes in extant invertebrates and a *BCL6*/*BCL6B* precursor gene in early vertebrates. The latter subsequently acquired additional sequences prior to duplication to yield distinct *BCL6* and *BCL6B* gene lineages that ultimately gave rise to the present-day mammalian genes. A subsequent teleost-fish-specific duplication yielded an additional *bcl6ab* gene.

## 3. Gene Expression

The expression of *BCL6* and related genes follows complex patterns throughout the lifespan of various organisms, with both immune and non-immune cell expression observed across different species, the latter notably including neurosensory tissues. During embryogenesis, zebrafish *bcl6* is expressed during the primitive wave of hematopoiesis in both the anterior and posterior lateral plate mesoderm, consistent with the location of early hematopoietic precursors [14]. Its expression continues into the definitive wave, with a strong expression in the thymus of developing zebrafish [14], consistent with the robust *Bcl6* expression observed in the fetal thymus and spleen of mice [18] and the *BCL6* expression observed in human fetal thymocytes [19]. In adults, pufferfish *bcl6* is expressed in the thymus as well as in the kidney, equivalent to teleost bone marrow [13]. The expression of mouse *Bcl6* was also identified in the thymus [18], with human *BCL6* expressed in peripheral blood leukocytes and lymph nodes [20]. Its strong expression was also noted across mouse and human pre-B and mature B cell lines, with a lower expression in T cell, myeloid, and erythrocyte lines derived from both humans and mice [21].

Outside of hematopoietic tissue, *bcl6* expression was observed in the embryonic retina of zebrafish [22] and in their developing cerebellum and medulla [14], and *Bcl6* expression was observed in the olfactory epithelium of prenatal mice [18], showing parallels with the expression of *ken* in fruit-fly cephalic furrows and larval eye antennae [17]. The expression of mouse *Bcl6* was additionally observed in their pre-natal skeletal muscle, esophagus, upper airway lining, and skin [18], whilst fruit-fly *ken* was also expressed in their developing gut [17]. In adults, pufferfish *bcl6* expression was identified in their skeletal muscle, intestine, ovary, brain, and nasal cavity [13]. Mouse *Bcl6* was similarly expressed within skeletal muscle as well as in the cerebral cortex [18,23], with human *BCL6* expression being described in adult skeletal muscle, thyroid, trachea, ovary, prostate, and spinal cord [20].

## 4. Structure and Function

**Figure 1:**
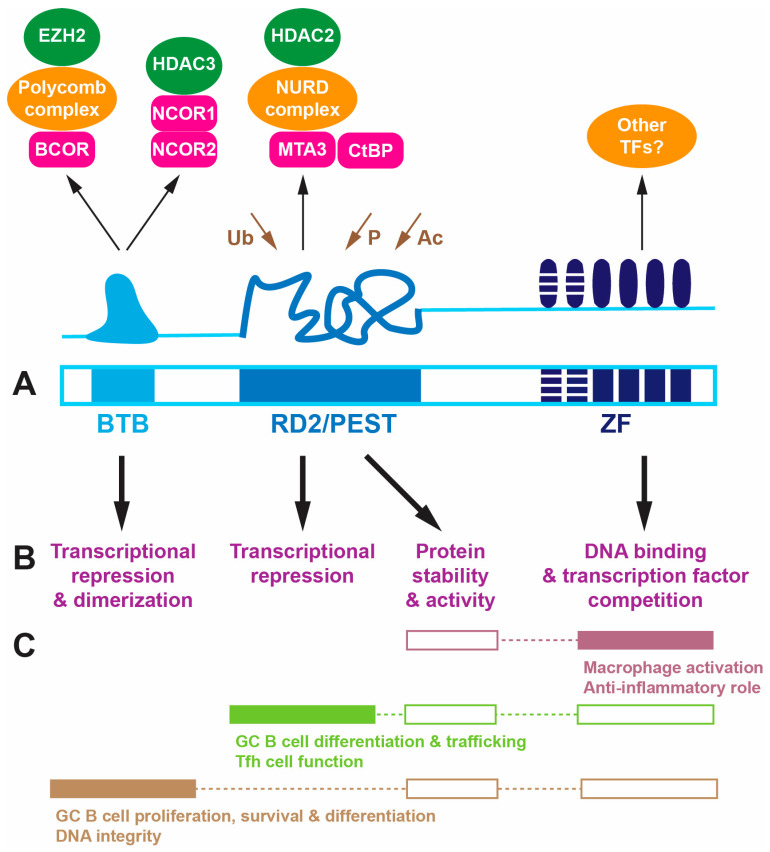
Structure and function of the BCL6 protein. (**A**). Domains of the BCL6 protein: BTB (light blue), RD2/PEST (blue), and ZF (dark blue, with striping for fingers not involved in DNA binding). Above is a schematic representation of the structure of each domain along with their interacting proteins, including co-repressors (pink) and associated transcriptional regulators (orange) and DNA modifying proteins (green), with sites of ubiquitination (Ub), phosphorylation (P), and acetylation (Ac) indicated (brown). (**B**). Major molecular function(s) of each domain. (**C**). Biological roles mapped to the molecular functions, with proven connections shown as filled boxes and assumed ones as unfilled boxes. Abbreviations: BTB: broad complex/tram track/bric-a-brac; RD2: repression domain 2; PEST: proline–glutamic acid–serine–threonine; ZF: zinc finger.

BCL6 is a 95 kDa multi-domain protein, which comprises a broad complex/tram track/bric-a-brac (BTB) domain at its N-terminus, a central unstructured region containing the so-called second repression domain (RD2) that overlaps with a proline (P), glutamic acid (E), serine (S) and threonine (T)-rich PEST domain, and a zinc finger (ZF) domain comprising an array of six C_2_H_2_ Krüppel-type zinc fingers at its C-terminus [24,25] (Figure 1A). An identical domain structure is seen across vertebrate BCL6 proteins [13,14], while Ken has fewer ZFs, with no RD2/PEST domain being identified [16].

The BTB domain facilitates dimerization as well as interactions with distinct sets of co-repressors [26]. This includes BCL6 co-repressor (BCOR), which associates with Polycomb complex proteins that, in turn, recruit histone demethylases such as enhancer of zeste homolog 2 (EZH2) [27] as well as nuclear co-repressor 1 (NCOR1) and NCOR2/SMRT, which principally associate with histone deacetylase 3 (HDAC3) [28]. RD2 sequences interact with alternative co-repressor metastasis-associated protein 3 (MTA3), which associates with NURD complex proteins to also recruit HDAC2 [29,30] and CTBP [31]. The PEST sequences controls protein stability, mediated by multiple ubiquitination sites in this domain. The disordered RD2/PEST domain also regulates activity via the acetylation of a lysine residue by p300, which can ablate the ability of BCL6 to repress transcription [32], as well as via phosphorylation by mitogen-activated protein kinases (MAPKs) and other kinases [33]. Within the ZF domain, the four C-terminal fingers facilitate binding to specific DNA binding sites related to a core 5′-TTCCTAGAA sequence [34], but this domain has also been implicated in protein–protein interactions [34], particularly with other transcription factors and potentially additional HDACs [35].

Thus, BCL6 functions principally as a transcriptional repressor via the deacetylation and/or demethylation of histones in the vicinity of its target sites [24]. However, it can also impact transcription by directly blocking access of other transcription factors to adjacent or overlapping target sites within the genome [36]. Through these mechanisms, BCL6 is able to actively repress many different genes depending on the cellular context, such as those involved in DNA integrity, cell cycle regulation, and differentiation [24,25,37]. Notably, key targets include a number of transcriptional repressors, including itself, with the ‘repression of repressor’ mechanism leading to the upregulation of many genes indirectly [5].

Indeed, a hallmark of BCL6 and its modus operandi is its interplay with other transcription factors in a range of regulatory networks. These regulatory networks are typically specific for their role in particular cell populations and are often antagonistic. For example, BCL6 shows reciprocal antagonism with the transcription factor B-lymphocyte-induced maturation protein 1 (BLIMP-1, also known as PRDM1) [38], which also shows evolutionary conservation, with homologues in zebrafish and fruit-fly. BCL6 and BLIMP-1 each act to repress the transcription of the other, which serves as a cell fate switch in the differentiation of a range of immune cell lineages [39]. BCL6 also has a reciprocal antagonistic relationship with STAT5, which is activated by various cytokines to directly repress *BCL6* transcription via binding to tetrameric sites in the *BCL6* promoter [11,40]. Conversely, BCL6 negatively regulates a subset of STAT5-responsive genes via overlapping DNA binding motifs [41]. This reciprocal regulatory relationship is conserved, with almost identical tandem tetrameric STAT5 binding sites found within the zebrafish *bcl6* promoter. Fruit-fly *ken* has also been demonstrated to be a target gene for its STAT homologue, Marelle (Stat92E) [42,43], while the Ken protein is able to block the expression of Stat92E target genes [44]. BCL6 also inhibits the actions of other transcription factors that drive differentiation down alternative pathways, such as TBX21, GATA3, and RORA [5].

## 5. Key Roles Played by BCL6

BCL6 has been shown to play important roles in the development and function of both lymphoid and myeloid immune cells, with additional roles outside the immune system.

### 5.1. B Cell Development and Function

BCL6 has been firmly established as a key regulator of GC B cells, which are fundamental for producing the high-affinity, class-switched antibodies essential for potent immunity [45]. BCL6 contributes to several key aspects of GC B cell development, including (i) promoting differentiation by suppressing genes such as *BLIMP1*; (ii) accommodating somatic hypermutation and class-switch recombination as part of the production of appropriate high-affinity antibodies, mediated by the repression of genes typically triggered by DNA damage, such as *TP53* and Ataxia telangiectasia and Rad3-related (*ATR*); (iii) stimulating proliferation important for clonal expansion, involving genes like cyclin-dependent kinase inhibitor 1A (*CDKN1A*); and (iv) enabling cell death as part of clonal selection via the repression of anti-apoptotic genes such as *BCL2* [3,24,37,46,47]. Bcl6-knockout mice failed to form germinal centers (GC) and produced high-affinity immunoglobulins associated with a distinct absence of GC B cells [2,48,49]. There was a significant reduction in bone marrow pre-B-cell self-renewal and differentiation [50], although plasma cell differentiation was maintained [51,52]. Interestingly, *BCL6* downregulation was needed for the establishment and maintenance of GC B memory cells [53]. Mice harboring either a BTB domain mutant [37,54] or RD2 domain mutant [55,56] showed defective GC B cell formation, highlighting the key role for gene repression in mediating the function of BCL6 in cells along this developmental pathway. Such studies revealed that repression of particular genes was specific to different domains. For example, the BTB domain regulated *ATR*, *TP53*, and *CDKN1A*, while the RD2 domain regulated *BLIMP1* [57].

### 5.2. T Cell Development and Function

BCL6 additionally plays a pivotal role in regulating the differentiation of specific T cell populations. In particular, it has been shown to drive the generation of Tfh cells, which facilitate antibody production by GC B cells, at the expense of other T helper (Th) subsets. Consequently, Bcl6-knockout mice displayed a significant decrease in the differentiation (and survival) of Tfh cells, with increased the differentiation of Th1 cells, which are involved in cell-mediated responses to intracellular infections, Th2 cells, which mediate humoral immune responses to extracellular parasites, and Th17 cells, which respond to extracellular infections at mucosal surfaces as well as tissue injury [2,4,58]. Key to this was the ability of BCL6 to inhibit key transcription factors driving the differentiation of Th1 (TBX21), Th2 (GATA3), and Th17 (RORA/RORC) cells [5]. Knockdown of Bcl6 in naive CD4+ T cells also resulted in increased differentiation of Th9 cells, which are involved in helminth infections and tumor immunity [59]. Conditional deletion of Bcl6 in T cells resulted in a 90% decrease in Tfh cells, with a 5-fold reduction in IgG, highlighting the impact on GC B cells [60]. BCL6 has also been shown to play a separate role in Tfh cell maintenance [61]. Interestingly, BTB mutant mice possessed normal Tfh cells [37,54], while RD2 mutants showed only partial impacts [55,56]. These studies revealed a more selective role for gene repression in the Tfh cell lineage, with direct competition with other transcription factors for DNA sites playing a significant role [8]. Other studies have identified additional functions for BCL6 in the generation of CD8+ central memory T (Tcm) cells, which underpin immune surveillance by lymph nodes [6], and the maintenance of long-term CD4+ memory T (Tm) cells, which provide a rapid response to previously encountered antigens [7], processes in which *BLIMP1* repression is important [39]. BCL6 also contributes to Treg stability and functionality [62] and also promotes the development of follicular regulatory T (Tfr) cells, a Treg subset that promotes antigen-specific over self-reactive B cell clones in the GC [63,64], as well as various innate-like T cells [65]. A significant decrease in embryonic T lymphocytes was also observed in Bcl6-deficient zebrafish embryos [14], demonstrating conservation of the BCL6-mediated regulation of T cell development, although the impact on specific subsets remains to be delineated.

### 5.3. Macrophage Development and Function

BCL6 has separately been implicated in the regulation of macrophage development and function. Macrophages from Bcl6-knockout mice showed significantly enhanced M1 polarization but decreased macrophage motility and spreading [10,66], concomitant with disrupted chemokine production [67]. Significantly reduced macrophage numbers and reduced motility in response to wounding were also observed in Bcl6-deficient zebrafish embryos, which also displayed an enhanced susceptibility that was likely due to these macrophage defects [14]. In addition, Ken disruption impacted fruit-fly macrophage-like hemocytes cells [17], collectively suggesting an evolutionarily conserved role in macrophage cells. BCL6 has also been implicated in the development and function of dendritic cell (DC) subpopulations [68,69,70].

### 5.4. Inflammation

One of the most prominent phenotypes displayed by Bcl6-knockout mice was severe Th2-mediated inflammation with distinctive eosinophilic infiltration, affecting the heart, lungs, liver, and spleen, leading to profound myocarditis and vasculitis [2,52,71]. This is a likely major contributor to their poor survival, with most Bcl6-knockout mice not surviving past 9 weeks of age and some dying as early as 3 weeks [2,52,71]. No inflammatory phenotype was evident in mice harboring either a Bcl6 BTB mutant [37] or Bcl6 RD2 mutant [55], indicating that the repressive function of Bcl6 does not play a significant role in this phenotype, which is instead mediated by the ZF domain. The ability of BCL6 to inhibit inflammation has been largely attributed to its role in suppressing the activation of NLRP3 inflammasomes in macrophages, with Bcl6-deficient murine macrophages showing enhanced NLRP3 activation, leading to a heightened production of key chemokines driving Th2 inflammation [67,72]. Zebrafish embryos deficient in Bcl6 also showed an enhanced expression of the pro-inflammatory cytokine Il1b, also thought to lie downstream of NLRP3, with similar poor survival [14].

### 5.5. Growth and Other Aspects of Development

BCL6 has been demonstrated to impact growth and development more broadly, with evidence that this also represents a conserved function. Bcl6-knockout mice exhibited significantly reduced postnatal growth [2,71] in concert with a severe decrease in adipose mass [73]. Juvenile Bcl6-deficient zebrafish showed a similar growth retardation and reduced adiposity [14]. The background dysregulation of immunity and inflammation has complicated the interpretation of these phenotypes. However, defective skeletal muscle differentiation was reported in Bcl6-deficient mice [18,23,71], with skeletal-muscle-specific Bcl6 ablation resulting in a 30% reduction in muscle mass, concomitant with a disruption in the expression of genes involved in proteostasis, suggesting a direct effect on this tissue [74]. Altered hepatic lipid metabolism, including reduced expression of lipogenic enzyme genes, has been observed in Bcl6-deficient mice [73], with BCL6 separately identified as a regulator of early adipose commitment in mesenchymal stem cells, controlling expression of early and late adipogenic regulators [75]. However, adipose-specific Bcl6 ablation instead resulted in a specific increase in inguinal (but not perigonadal) adipocyte size and mass, attributed to altered insulin sensitivity and the prevention of steatosis [76].

BCL6 has also been identified as a pro-neurogenic factor during embryonic neocortex development [77], playing roles in progenitor cell differentiation [78] as well as in the survival of cortical neurons [79] and olfactory sensory neurons [80]. The ablation of Ken impacted behavioral responses to visual stimuli in fruit-flies, including escape behavior and synaptic function in the giant fiber system [81], suggesting some potential conservation. The knockdown of zebrafish Bcl6 was separately implicated in aberrant optic cup formation during embryogenesis [22], although this appeared to be unaffected in Bcl6-deficient zebrafish or Bcl6-deficient mice.

Finally, BCL6 can independently impact various aspects of reproduction. Bcl6-knockout mice showed 80% lower spermatozoa at 8 weeks of age, with only 15% being fertile, which correlated with enhanced spermatocyte apoptosis [82]. Ken has also been shown to maintain the self-renewal capacity of testis cells [44] and the proper development of external genitalia [16]. BCL6 has separately been shown to contribute to trophoblast migration/invasion during early placental development [83].

## 6. Role in Malignancy

The role of BCL6 in the etiology of cancer is complex. It can act as an oncoprotein in many types of cancer, but there are also examples where it performs a tumor-suppressor function. It is able to additionally influence tumor immunity that can be either promote or inhibit tumorigenesis. Finally, BCL6 facilitates therapy resistance across a range of cancer types.

### 6.1. Oncogenic Functions

*BCL6* was first identified as a frequent target gene for chromosomal translocations at chromosome 3q27 in NHL, particularly diffuse large B cell lymphoma (DLBCL) and follicular lymphoma (FL) [1,84], but it was subsequently found to be overexpressed by this and other mechanisms in a range of B cell malignancies extending across B cell lymphomas and leukemias [85]. It plays particularly key roles in more indolent forms of NHL, including FL, the DLBCL subtype primary mediastinal B cell lymphoma (PMBCL), as well as in intravascular large B cell lymphoma (IVLBCL), an aggressive high-grade B cell lymphoma that lies intermediately between DLBCL and Burkitt’s lymphoma [86,87]. It also forms part of the criteria for the revised WHO classification for high-grade B cell lymphomas, defined as those with mutations in *MYC* in concert with either *BCL6* or *BCL2*, which show poor responses to standard therapy [88]. In the majority of cases, an increased *BCL6* expression is mediated by translocations involving fusions of strong promoters active in B cells, such as the heavy and light immunoglobulin chain loci, to the BCL6 gene, leading to deregulated expression [89]. Additionally, deletions and other mutations are common in the 5′ non-coding exon disrupting auto-regulatory BCL6-binding sites [90] and/or repressive STAT5-binding sites [11], leading to enhanced *BCL6* expression. In addition, mutations that disrupt factors involved in *BCL6* regulation have been identified in B cell malignancies [91]. At the transcriptional level, these include activating mutations in the major positive regulator of BCL6, myocyte enhancer factor 2B (MEF2B) [92], and inactivating mutations in pathways involved in its repression, such as interferon regulatory factor 8 (IRF8) [93]. Post-translationally, this includes inactivating mutations in proteins involved in its acetylation, including CREBBP and EP300 [94], and ubiquitination, such as F box only protein 11 (FBXO11) [95]. Alternatively, enhanced upstream pathways can be involved. For example, BCL6 is highly expressed in B cell acute lymphoblastic leukemia (ALL) mediated by pre-BCR signaling [96] and Ikaros [97]. Regardless of the mechanism, high *BCL6* expression ensues, resulting in an enhanced expression of oncogenic genes such as *BCL2*, *MYC*, *CCND1*, and *BMI1* to facilitate lymphomagenesis [98].

BCL6 has additionally been identified as a driver of other hematological malignancies [25]. *BCL6* is expressed at high levels in acute myeloid leukemia (AML) cell lines and primary AML cells [99], including aggressive AML [100]. It has further been shown to maintain the survival and self-renewal of primary AML by maintaining stem/progenitor cells [99]. It has additionally been demonstrated to facilitate leukemia initiation and self-renewal in chronic myeloid leukemia (CML) [101]. BCL6 also potentially contributes to HTLV-1-mediated adult T cell leukemia by enhancing cell cycle progression [102].

BCL6 has been implicated as an oncoprotein in solid tumors as well. In these cases, it is typically highly expressed in cancer cells, facilitating the enhanced expression of genes involved in proliferation, such as that encoding the cell cycle regulator cyclin D1 (*CCND1*), and decreasing genes involved in monitoring DNA integrity/repair, notably including *TP53* [25]. Thus, *BCL6* has been found to be highly expressed in breast cancer and associated with enhanced disease progression and decreased patient survival, with its overexpression increasing tumor growth and invasion in a xenograft model [103]. In ovarian cancer, high *BCL6* expression correlated with high tumor burden [104] and poorer prognosis [104,105], with similar associations identified in the context of high-grade glioma [106]. However, the mechanisms involved in the enhanced expression are varied. In glioblastoma, *BCL6* represents a key downstream target of MED12, with the increased BCL6 protein able to suppress TP53-mediated apoptosis [107], while it lies downstream of KRAS in lung cancer [108]. In colorectal carcinoma, hypoxia-induced long non-coding RNA (lncRNA) 00205 was shown to bind microRNA (miR)-10a and miR-34c, relieving their suppression of *BCL6* mRNAs, with the elevated BCL6 protein then able to block TP53-mediated gene repression, thereby promoting metabolic changes that enhanced cancer progression [109]. In contrast, an increased *BCL6* copy number has been observed in squamous cell carcinoma of the lower gastrointestinal tract [110]. Finally, a mechanism involving direct stabilization of the BCL6 protein by the tumorigenic (lncRNA) 00152 has been identified in ovarian cancer [111].

### 6.2. Tumor-Suppressor Functions

There is a growing recognition that in certain cancer types BCL6 instead functions as a tumor suppressor. Thus, in nasopharyngeal carcinoma, either a reduced *BCL6* expression or the presence of a *BCL6-SPECC1L* fusion, which removes the repressive function of the protein, has been associated with enhanced growth [112]. It also suppressed the development of medulloblastoma in a mouse model through repression of the sonic hedgehog pathway [113]. In gastric cancer, a significantly decreased *BCL6* expression has been observed, with a low expression associated with a more malignant clinical phenotype and poor patient prognosis [114]. BCL6 has also been shown to suppress non-alcoholic steatohepatitis (NASH)-induced liver injury associated with hepatic cancer [115].

### 6.3. Therapy Resistance

BCL6 has been separately demonstrated to inhibit the effectiveness of cancer therapies, with BCL6 often found to be upregulated by the therapeutic agent. This was originally described in the context of B cell lymphoma cell lines [116] but has subsequently been shown to be responsible for resistance to a broad range of inhibitors across multiple cancer types. Thus, BCL6 has been associated with resistance to HDAC inhibitors in DLBCL [117], BCR-ABL1 kinase inhibitors in ALL [118], cytarabine in AML [99], paclitaxel in breast cancer [119], and BET inhibitors in KRAS+ non-small-cell lung cancer [120]. *BCL6* was induced by imatinib in gastrointestinal stromal tumor (GIST) [121]. It was also induced by epidermal growth factor receptor (EGFR) inhibitors in non-small-cell lung cancer (NSCLC), resulting in reduced apoptosis [122], while in glioblastoma, either chemotherapy or radiotherapy could upregulate *BCL6* expression, which facilitated a blunting of the effectiveness of these therapies [123]. It has been suggested that this role for BCL6 is part of an evolutionarily conserved stress response that enables cancer cells to adapt to stressors, including therapeutic agents [124]. This involves the induction of *BCL6* via the HSF-1 stress response, which is, in part, mediated by de-repression of the *TOX* gene [125]. However, there is evidence for other mechanisms inducing *BCL6*. For example, in CML, *BCL6* upregulation by kinase inhibitors has been shown to be facilitated by interferon-gamma-induced STAT1 activation [126] and the relief of STAT5-mediated *BCL6* gene repression [118]. Other studies have shown that *BCL6* expression impacts the transcriptome in a similar manner to normal cells, such as the repression of *TP53*, leading to suppressed apoptosis [121].

### 6.4. Tumor Immunity

Distinct from its other roles in cancer cells, BCL6 separately influences tumor immunity in a variety of ways. BCL6 has been shown to promote the development and maintenance of tumor-associated stem/progenitor-like CD8+ T cells associated with the persistence of anti-tumor responses [127], providing sustained anti-tumor immunity in the context of lung cancer [128] and melanoma [128,129]. Conversely, BCL6 can also preserve the ability of Treg cells to restrict effector T cell function in the tumor microenvironment, with enhanced Treg expression of *BCL6* correlating with poor prognosis in colorectal cancer and melanoma lymph node metastasis [130], while BCL6 has also been shown to suppress the infiltration of CD4+ T cells to promote hepatocellular carcinoma development [131]. Finally, BCL6 is able to induce a stem-like memory program in tumor-associated macrophages to promote long-lasting pro-tumor immunity [132].

## 7. BCL6-Based Therapeutics

The central role played by BCL6 in various aspects of biology and disease has made it an attractive target for therapeutic intervention. A number of therapeutic agents have been developed, which have been comprehensively reviewed [133]. The majority of these are small molecules that reversibly associate with the BTB domain to inhibit its interactions with co-repressors [134]. These include FX1 [135], WK369 [136], WK499 [137], 79-6 [138], CCT369260 [139], and the orally-available WK500B [140] and GSK137 [141], while FX1 has also been formulated as a pro-drug, AP-4-287, to improve water solubility [142]. TMX-2164 also targets the BTB domain but reacts covalently with a tyrosine residue to act as an irreversible inhibitor [143]. In addition, other molecule classes have also been developed, including an aptamer-based Apt48 [144] and a retro-inverso peptide inhibitor RI-BPI [145]. Other therapeutics targeting this domain act by promoting the proteasomal degradation of BCL6 via the ubiquitination pathway, either small molecules triggering polymerization [146] or so-called proteolysis targeting chimera (PROTAC) approaches that recruit E3 ligases [147]. Alternatively, siRNA [148] and miRNA [149] targeting *BCL6* transcripts have also been developed.

BCL6 inhibitors have proven efficacious in a number of disease contexts. These include targeting the direct oncogenic role played by BCL6 in hematological malignancies. Efficacy in this context has been demonstrated for FX1 [135], RI-BPI [145], and WK500B [140] in BCL6-dependent DLBCL xenograft models. Such agents have also proven effective in the context of AML, with RI-BPI increasing apoptosis and reducing stem-ness [99] and WK500B inducing cell cycle arrest and apoptosis [137]. The utility of BCL6 inhibitors extends to solid tumors, with WK369 being shown to inhibit the growth and metastasis of ovarian cancer [136]. In a novel approach, the introduction of miR-144-3p, which targets *BCL6*, was able to suppress the proliferation and invasion of glioma cells [149]. Inhibitors have also been effective in situations where BCL6 mediates resistance to therapy. BI-3802 was shown to restore/enhance the effectiveness of imatinib, promoting increased apoptosis in the setting of GIST [121,150], and FX1 overcame resistance to the JAK1/2 inhibitor ruxolitinib in CRL2-rearranged ALL [151] and synergized with the EGFR inhibitor gefitinib in NSCLC [122], while WK499 synergized with chemotherapy in ALL [137]. Clinical trials are currently underway to evaluate PROTAC-based BCL6 inhibitors in the context of relapsed/refractory NHL, such as ARV-393 (Arvinas, phase 1, NCT06393738) and BMS-986458 (Bristol-Myers-Squibb, phase 1/2, NCT06090539).

Alternatively, BCL6 inhibitors have been utilized in suppressing the normal role of BCL6 in immunity, such as in the settings of immune rejection and autoimmunity. For example, the BCL6 inhibitor 79-6 protected against graft-versus-host disease in an allogeneic hematopoietic stem cell transplantation model, with a concomitant reduction in Tfh cells [138]. FX1 prolonged the long-term survival of cardiac grafts concurrently with a reduction in Tfh and other T cell populations [152]. This agent has also been shown to ameliorate symptoms in a mouse model of lupus-like autoimmunity through attenuating the Tfh, GC B, and Th1 cell responses [153]. Finally, an siRNA targeting *BCL6* was able to ameliorate asthma symptoms in a mouse model [148].

## 8. Conclusions

The knowledge base regarding BCL6 is very large, with its functions in lymphoid cells and lymphoid malignancies being understood in intricate detail, often establishing key paradigms in their relevant fields. However, there is still much to learn, including the roles played by BCL6 across innate immune cell populations and in other aspects of biology, as well as the transcriptional networks it influences, extending to those involving BCL6B and other so-called ZBTB proteins. Deeper understanding is also required regarding the role of BCL6 in the non-malignant diseases where it has been implicated, such as pre-eclampsia [154], endometriosis [155], and ischemic stroke [156]. Moreover, the multiple roles played by BCL6 mean that therapeutic approaches need to be developed with an abundance of caution to ameliorate potential adverse effects. For example, in B cell lymphoma, the use of BCL6 inhibitors relieved the BCL6-mediated repression of *BCL2*, leading to addiction to this oncogene [157]. Moreover, in a mouse bronchopulmonary dysplasia model, the FX1 inhibitor was shown to worsen the pathology, which was attributed to the role of BCL6 in inhibiting inflammation [158]. Therefore, the continued advancement of therapeutic agents allowing for the precise manipulation of BCL6 depending on the disease context is essential.

## Data Availability

No new data were created or analyzed in this study. Data sharing is not applicable to this article.
